# The BLISS cluster randomised controlled trial of the effect of 'active dissemination of information' on standards of care for premature babies in England (BEADI) study protocol [ISRCTN89683698]

**DOI:** 10.1186/1748-5908-2-33

**Published:** 2007-10-08

**Authors:** Dominique Acolet, Kim Jelphs, Deborah Davidson, Edward Peck, Felicity Clemens, Rosie Houston, Michael Weindling, John Lavis, Diana Elbourne

**Affiliations:** 1 Medical Statistics Unit, London School of Hygiene and Tropical Medicine, Keppel Street, London WC1E 7HT, UK; 2 The Confidential Enquiry into Maternal and Child Health (CEMACH) Central Office, Chiltern Court, 88 Baker Street, London NW1 5SD, UK; 3 Health Services Management Centre, University of Birmingham, Park House, 40 Edgbaston Park Road, Birmingham B15 2RT, UK; 4 Health Sciences Centre, McMaster University,1200 Main St. West Hamilton, ON L8N 3Z5, Canada

## Abstract

**Background:**

Gaps between research knowledge and practice have been consistently reported. Traditional ways of communicating information have limited impact on practice changes. Strategies to disseminate information need to be more interactive and based on techniques reported in systematic reviews of implementation of changes. There is a need for clarification as to which dissemination strategies work best to translate evidence into practice in neonatal units across England. The objective of this trial is to assess whether an innovative active strategy for the dissemination of neonatal research findings, recommendations, and national neonatal guidelines is more likely to lead to changes in policy and practice than the traditional (more passive) forms of dissemination in England.

**Methods/design:**

Cluster randomised controlled trial of all neonatal units in England (randomised by hospital, n = 182 and stratified by neonatal regional networks and neonatal units level of care) to assess the relative effectiveness of active dissemination strategies on changes in local policies and practices. Participants will be mainly consultant lead clinicians in each unit. The intervention will be multifaceted using: audit and feedback; educational meetings for local staff (evidence-based lectures on selected topics, interactive workshop to examine current practice and draw up plans for change); and quality improvement and organisational changes methods. Policies and practice outcomes for the babies involved will be collected before and after the intervention. Outcomes will assess all premature babies born in England during a three month period for timing of surfactant administration at birth, temperature control at birth, and resuscitation team (qualification and numbers) present at birth.

**Trial registration:**

Current controlled trials ISRCTN89683698

## Background

Patient care often does not take into account new research findings. Studies in the United States suggest that the care of 30 to 40% of patients is not based on up-to-date scientific knowledge [1,2]. Health care research consistently finds a gap between research evidence and actual practice in the delivery of care [3]. The main conclusions from a large systematic review on interventions to disseminate information and change clinical practice were a limited or mixed effect of the traditional more passive ways used by health professionals to keep up-to-date with their practice (educational material, conferences and courses) [3]. Even if a practitioner is aware of new findings, barriers to change may hamper local changes in practice [3]. There is growing evidence of the effectiveness of different interventions to bring change in clinical practice [4-6], and their knowledge may help to design dissemination strategy [3].

### Relevant literature

A systematic review [7] of the main individual interventions studied showed that the most promising methods were: continuing medical education activities based on 'interactive workshops' [8]; educational outreach by experts or trained facilitators, referred to as academic detailers ('face to face visit') [9,10]; use of local opinion leaders ('champions') [11,12]; audit and feedback especially when baseline adherence to recommended practice is low [13], and use of reminders [14,15]. The effect of any of these interventions considered separately on policy and practice changes are modest to moderate (10 to 15%) [3]. Combined interventions (multifaceted interventions) aiming at acting on different levels of barriers to change may be more effective than individual interventions [3].

There is nevertheless imperfect evidence to support decisions regarding strategies that are likely to be appropriate and effective under varying circumstances [15] and considerable judgement is required in the choice of intervention(s) to influence changes [15]. Which dissemination strategies work best in a setting such as neonatal care in England therefore need further clarification. One group has been working previously on the effect of active dissemination of neonatal research in the USA (Vermont Oxford Network) using data collected by a collaborative network of selected neonatal units [16]. They published a multifaceted collaborative quality improvement intervention to promote evidence-based surfactant treatment for preterm infants born at 23–29 weeks' gestation [17]. The study design was a cluster randomised controlled trial (CRCT). The intervention comprised audit and feedback, lectures on reviews of the evidenced-based literature, an interactive training workshop and ongoing faculty support via conference calls and email. This package of intervention was associated with a significant improvement (40%) in the process of care leading to earlier surfactant administration to improve survival in preterm infants of an order of magnitude much higher than the general 10–15% effect described in the literature. Possible explanations for this could be: a more targeted audience of clinicians working within a well defined subspecialty (neonatology); the enrolment of a tight network of clinicians working together and receptive to quality improvement and benchmarking of their performance [17]; a two-day interactive workshop based on a collaborative improvement initiative [17,18]; social networking during the meeting, which has been shown to contribute to the success of collaborative initiatives [19]; interactive networking with good communication between the main centre collecting evidence and detailing it proactively to the different hospitals in the network which has been shown in a recent systematic review to increase the prospects for research use among policy-makers [20]; and the use of the continuous quality improvement concept applied through the Rapid Cycle Improvement Process (RCIP) introduced in the Vermont Oxford North American Neonatal Network [16] by Paul Plsek [18,21].

Parallel work from the Health Services Management Centre (HSMC) in the UK has built on the organisational development cycle [22]. The main approaches, models and conceptual frameworks have been applied in a number of settings in the NHS and partner agencies [23]. The theoretical approach informs the practical process of planned change and provides a framework to introduce other research and theories focussed on leading and managing change and transition. The approach recognises and uses participants' experiential learning and employs metaphors to elicit experiences of change [24]. Other useful models include: the importance of recognising transition through exploring [25-27]; the emotions associated with the human dimensions of change including loss [28]; and an individual's personal capacity for change [29]. The theoretical and experiential approaches may be enhanced by practical hints, tips, and tools for local use. The choice of active participants in the change process may be crucial. In addition to the role of opinion leaders (often as classified by peers), employee participation theory suggests self-nominees (volunteers) may enhance local communications and coordination, employee motivation, and employee capability [30-33] in the change process.

### The case study

The UK Confidential Enquiry into Maternal and Child Health (CEMACH) has a nationwide network for data collection and assesses standards of care in a wide range of perinatal clinical areas. One CEMACH study, Project 27/28, reported variations in standards of care that might have contributed to the death of preterm babies born at 27 or 28 weeks' gestation [34]. Gaps found between evidence and practice led to the development of recommendations for future practice [34]. As a consequence, a new national position statement on the early care of premature babies has been developed by the UK's leading national institution for clinical governance in neonatology, the British Association of Perinatal Medicine (BAPM) [35]. Based on the literature described above, it was felt unlikely that a similar approach to disseminating these new recommendations alone would have major effects on new policies and practices at local hospital level. An attempt to measure the impact on policy and practice of a previous CEMACH report in 1998 concluded that frontline staff rarely have consistent access to the written report, and that internal dissemination was often faulty [36]. One of the recommendations made to improve dissemination was the production of video/audio materials (educational material) for professional development meetings to be sent to a single lead disseminator [36]. As a consequence, a "dissemination package" of the main findings and recommendations was sent to each Trust in England, Wales and Northern Ireland (a PowerPoint presentation to inform discussion at local hospital clinical governance meetings).

As an attempt to evaluate the impact on policy and practice of the main Project 27/28 report and the usefulness of the slide dissemination package, CEMACH sent a questionnaire to key potential UK recipients. Responses were received from 94 out of 262 neonatal/paediatric clinicians (36%), and 86 out of 183 acute Trusts with maternity services nationally (47%). Not all respondents answered all questions. Approximately three-quarters of the sample said they recalled receiving the dissemination package, and most of these reported using the slide package, finding it useful for raising awareness of the clinical issues and fostering the initiation and/or consolidation of policy and practice changes. They particularly appreciated the presentations specifically tailored for different audiences.

Although these responses suggested that dissemination initiatives might be helpful, it was difficult to draw firm conclusions from the poor response rate. Therefore, we felt that a more scientifically robust evaluation of innovative strategies for the dissemination of information would be needed [37] to improve knowledge transfer leading to policy and practice changes in the care of premature babies in England.

### Objective

The main aim of this study will be to use the rigour of a randomised controlled trial in an evaluation comparing the effects of different approaches on policy and practice in the care of preterm babies in England. The specific objective will be to assess whether an innovative 'active' strategy for the dissemination of neonatal research findings, recommendations, and national neonatal position statement is more likely to lead to changes in policy and practice than a more passive form of dissemination involving just circulating the 27/28 Report, sending the dissemination slide package to hospital staff, and making the guidelines available on the website.

## Methods/design

When the intended effect is practice and policy changes at an institutional level, cluster randomised controlled trials (CRCTs) where randomisation is by hospitals allowing the delivery of the intervention to be focused on the whole staff is the most appropriate design [37]. We will therefore conduct a CRCT (randomised by hospital) to assess the relative effectiveness of dissemination strategies. The findings of Project 27/28 and particular aspects of the new British Association of Perinatal Medicine (BAPM) position statement will be used as the case study.

### Participants

**Figure 1:**
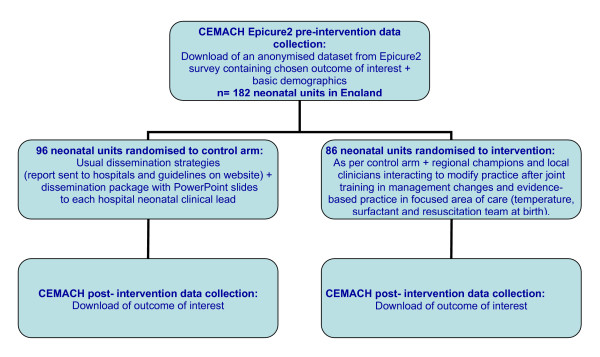
Flow chart of the CRCT.

The main participants in the BLISS cluster randomised controlled trial of the Effect of 'Active Dissemination of Information' on standards of care for premature babies in England (The BEADI Study) will be clinicians from neonatal units, although data will also be collected about premature babies. All neonatal units in England (182 hospitals in England with neonatal intensive care facilities for premature babies) were identified by CEMACH at the beginning of 2006 (Fig 1). Neonatal units have been randomised to the active arm or control (Fig 1) and the randomisation process stratified by neonatal networks (n = 25) based in different health regions and by units' level of care delivered (level one to three). Some hospitals designated as level two to three or 2.5 were classified as level two. To allow randomisation to be reproduced within each strata, data were ordered by network and level of care in ascending order and then by name of hospital by alphabetic order. Data from Excel dataset was imported into statistical computer software Stata 9. Stata 9 does not directly allocate a treatment (active arm or passive) within each stratum, but generates a list of block stratified randomisation code, and within each block it allocates a treatment at random. The programme generates a series of blocks of varying size (two, four, or six) for each stratum and then allocates treatment randomly within each block. Because the number of hospitals within each block is variable, some of the treatment codes (allocation to active or passive treatment) were not used. The unused allocations within each stratum were discarded. This process was likely to generate some allocation imbalance. Among the 182 hospitals enrolled in the Epicure2 study that were randomised, 86 were allocated to the active arm and 96 to the control group (Fig 1).

Clinicians from the neonatal units randomised to the active arm will be approached and asked to volunteer to play one of the following roles:

1. Regional 'champions', based in areas in which there are units which have been randomised to the intervention arm, and who will be recruited by CEMACH, given the relevant information, and asked to attend the first and second intervention meetings and provide ongoing support to local clinicians (see Interventions, below);

2. Clinicians in units which have been randomised to the intervention arm will also be nominated by CEMACH, given the relevant information, asked to attend the second intervention meeting only, and to then work at implementation of the BAPM guidelines in their local unit (see Interventions, below).

All babies born at < 27 weeks' gestation in England during the study period will be identified using the dataset of another national study on premature babies running at the same time as the BEADI study [38], Epicure2, which investigates survival and long term outcomes of premature infants below 27 weeks' gestation in England in 2006 compared to the outcome in 1995 [39].

### Intervention

Overall, the intervention design will be based on intervention(s) aimed at groups of specific health professionals that have been shown to be effective in isolation or in association (multifaceted) in adult medicine and in particular in UK, and neonatal care in a North American context (see Background), [3,8,11,17,18,30-33].

The intervention process will include two meetings (Fig 1):

1. At the first meeting, the regional 'champions' will come together: to explore the theory and practice of NHS organisational change; to consider the role of champions as leaders; to understand behaviour change principles and the human dimension of changes, and to develop practical skills to effect and sustain change in order to support health care staff in their workplace. They will be supervised by trainers with expertise in organisational change.

2. At the second meeting, the regional champions and consultant/senior nurses/leads for clinical governance from each intervention unit will then come together to: explore clinical areas identified for changes (with evidence based lectures from national clinical leaders); understand benchmarking of individual policies and practices; and to be introduced to tools for achieving changes in practice. They will then be asked to determine actions required to develop responses to suggested areas of changes locally and to support the processes needed to achieve these changes. They will also be supervised by the same trainers at the first meeting, who are experts in organisational change.

The control arm will be based on the current dissemination strategies (report sent, guidelines on website), which includes a dissemination package with PowerPoint slides (Fig 1).

### Choice of outcomes

The primary policy and practice outcomes must meet certain criteria. First, they need to be important for babies. Also, they must be able to be affected by implementing interventions that are evidence-based. Finally, the outcomes must be likely to be affected by an active dissemination intervention (*i.e*. not already used so extensively that there is no capacity for increased implementation). Two 'practice' outcomes that fulfil these criteria have been identified. First, the timing of surfactant administration at birth: Project 27/28 reported delays in surfactant administration in over 40% of cases [34,40] that were amenable to change in the US trial [21] of active dissemination strategies. Second, the temperature control of premature babies at birth: Project 27/28 showed that poor thermal control was strongly associated with death [34,40] and hypothermia may be prevented easily by using polyethylene occlusive skin wrapping to prevent heat loss in labour ward as soon as the premature baby is born [41]. Qualification and number of paediatric staff present at the delivery of a preterm infant has been identified as a 'policy' outcome as Project 27/28 reported 45% of inadequate staff cover at the time of the initial resuscitation of these babies at birth [34].

All these policy and practice outcomes fulfil the first and second criteria mentioned above, but there is currently only anecdotal evidence to inform the third criterion about the extent of use of these practices and policies. Therefore, data to quantify the pre-intervention extent of these policies and practices will be collected for each individual unit and baby in the study (Fig 1). Rather than setting up new national data collection, the BEADI study will be collaborating with a related study [38] also working with CEMACH and will be given an anonymised download from the Epicure2 dataset (Fig 1). Post-intervention data will be collected in the same way but additional data will be collected by CEMACH within three months after the intervention takes place to assess any trends in the outcomes over time (Fig 1). Both data collections process will be blind to the allocation intervention.

### Power calculation

The power calculations for assessment of the policy outcomes are based on the number of available hospitals known at the time [42]. Working backwards from an estimated 130 hospitals in England with neonatal intensive care facilities for premature babies that have been enrolled in the Epicure2 study, and considering a likely range of percentages which may have already implemented the relevant policies in the control hospitals (p1) (the rates for the outcomes of interest will not be known until analysis of the pre-intervention survey), we have calculated what size of effect could be detected with 80% power at 5% level of statistical significance (two-sided test), given the constraint of this fixed number of hospitals. For example, 126 hospitals are required to detect a change of policy from 60% in the control arm (p1) to 82% in the intervention arm (p2) with a size of effect (RR) of 1.4, irrespective of the number of babies (Table 1). Similarly, the power calculations for assessment of changes in practice outcomes are based on the number of babies clustered in the 130 hospitals. According to estimates from Epicure [39], one can expect 1650 annual admissions to neonatal intensive care from 3,500 births of babies < 27 weeks in England. As the data collection is based on fixed three month periods, the number of babies we can expect over three months is approximately 400 admissions and 850 births. Again, working backwards from numbers available, and additionally making assumptions about intra-cluster correlation coefficients (from published databases of likely intra-cluster correlation coefficient (ICC) in active dissemination research in previous trials), the trial is likely to have enough power to detect a range of practice changes. For example, 400 admissions will have around 80% power to detect a difference in practices from 40% to 55% (5% two-sided significance) with ICC of 0.06, and 850 births will have more than 80% power to detect a same difference even for an ICC of 0.25 (Tables 2, 3, 4).

**Table 1:** Power calculation for policies assessment

**% with policy in control arm (p_1_)**	**% with policy in intervention arm (p_2_)**	**Size of effect (RR)**	**Total number of hospitals needed to detect with 80% power at 5% level of statistical significance (two-sided test)**
60	82	1.4	126
55	78	1.4	124
50	74	1.5	121
45	69	1.5	126
40	64	1.6	128
35	59	1.6	128

**Table 2:** Power calculation for changes in practice for various assumptions of % with practice pre-intervention – p_1_(20–60%), p_2 _(30–90%), assuming 80% power at 5% level of statistical significance (2-sided test), for sizes of effect ≤ 2 and assuming no clustering (ie ICC = 0.00)

**Total number of babies needed**														
**p_1_**	**P_2_**													

	**30**	**35**	**40**	**45**	**50**	**55**	**60**	**65**	**70**	**75**	**80**	**85**	**90**	
**60**									712	304	162	98	62	
**55**								752	324	176	108	70	48	
**50**							774	338	186	116	76	54	38	
**45**						782	346	192	120	82	58	42	32	
**40**					774	346	194	122	84	60	44			
**35**				752	388	192	122	84	62					
**30**			712	324	186	120	84							
**25**		656	304	176	116									
**20**	546	276	162											

**Table 3:** Power calculation for changes in practice for various assumptions of % with practice pre-intervention – p_1 _(20–60%), p_2 _(30–90%), assuming 80% power at 5% level of statistical significance (2-sided test), for sizes of effect ≤ 2 and for cluster size three (numbers of babies admitted per hospital)

**ICC assumption required for 390 babies**														
**p_1_**	**P_2_**													

	**30**	**35**	**40**	**45**	**50**	**55**	**60**	**65**	**70**	**75**	**80**	**85**	**90**	
**60**										0.14				
**55**									0.01	0.6				
**50**								0.07	0.54					
**45**							0.06	0.5						
**40**						0.06	0.5							
**35**					0.07	0.5								
**30**				0.1	0.54									
**25**			0.14	0.6										
**20**		0.2												

**Table 4:** Power calculation for changes in practice for various assumptions of % with practice pre-intervention – p_1_(20–60%), p_2 _(30–90%), assuming 80% power at 5% level of statistical significance (2-sided test), for sizes of effect ≤ 2 and for cluster size six (numbers of annual live births per hospital)

**ICC assumption required for 780 babies**														
**p_1_**	**P_2_**													

	**30**	**35**	**40**	**45**	**50**	**55**	**60**	**65**	**70**	**75**	**80**	**85**	**90**	
**60**									0.02	0.31				
**55**									0.28					
**50**								0.26						
**45**							0.25	0.6						
**40**						0.25	0.6							
**35**				0.01	0.26	0.6								
**30**			0.02	0.28										
**25**		0.04	0.31											
**20**	0.06	0.36												

After completing the power calculations, further relevant hospitals were identified via the Epicure2 study [38] (increasing the sample from the estimated 130 to 182 hospitals). The power calculations are therefore conservative.

### Analysis

Statistical analysis of the RCT will be based on Intention to Treat (ITT) principles, comparing outcomes from all the hospitals allocated to active intervention with those allocated to control. Both for policies and for practice outcomes, the emphasis will be on differences between these groups post-intervention, and on differences between these groups in terms of changes between the pre-intervention and post-intervention phases when data are available pre-intervention. For the policies, this will be based on hospitals, but for practice outcomes, this will be based on babies within hospitals, taking appropriate account of the clustering.

### Ethical considerations

The approach and recruitment process for RCTs involving clusters (and especially involving educational interventions) is recognised to be different from that involving randomising individuals, and is closer to Zelen randomisation [43,44] in that randomisation comes before consent, and consent to intervention is usually only asked from those allocated to the active intervention arm. Information about BEADI will also be made available on the BAPM website [23]. Selected data items will be anonymously downloaded to CEMACH from the Epicure2 study (approved by Multi-centre Research Ethics Committees (MREC)- East London and the City Research Ethics Committee 2005) for the purpose of the BEADI study. Anonymised data will be stored securely within CEMACH indefinitely as per the CEMACH Information Security Policy. Any data identifying clinicians who have agreed to participate will only be stored by the trial team for the duration of the study and subsequent analysis, and will be anonymised for reporting. MREC approval was awarded for the BEADI study by the East London and the City Research Ethics Committee on 17 November 2005. A subsequent qualitative study on barriers to changes is planned and will be part of a separate protocol and submission to MREC.

## Competing interests

The author(s) declare that they have no competing interests.

## Authors' contributions

DA and DE had the original idea for the study. DA prepared the draft of the protocol in cooperation with DE, JL and MW. FC and RH provided a significant input at the planning phase of the study. KJ, DD and EP helped in the concept and design of the workshop/intervention days of the study. Trial randomisation was carried out by DA, FC and DE at the Medical Statistics Unit (LSHTM). All authors read and approved the final manuscript.
